# Structural Characterisation of Predicted Helical Regions in the *Chironex fleckeri* CfTX-1 Toxin

**DOI:** 10.3390/md16060201

**Published:** 2018-06-07

**Authors:** Athena Andreosso, Paramjit S. Bansal, Michael J. Smout, David Wilson, Jamie E. Seymour, Norelle L. Daly

**Affiliations:** Centre for Biodiscovery and Molecular Development of Therapeutics, Australian Institute of Tropical Health and Medicine, James Cook University, Cairns, QLD 4878, Australia; athena.andreosso@my.jcu.edu.au (A.A.); paramjit.bansal@jcu.edu.au (P.S.B.); michael.smout@jcu.edu.au (M.J.S.); david.wilson4@jcu.edu.au (D.W.); jamie.seymour@jcu.edu.au (J.E.S.)

**Keywords:** *Chironex fleckeri* venom, CfTX-1, cubozoan toxins, pore-forming toxins

## Abstract

The Australian jellyfish *Chironex fleckeri*, belongs to a family of cubozoan jellyfish known for their potent venoms. CfTX-1 and -2 are two highly abundant toxins in the venom, but there is no structural data available for these proteins. Structural information on toxins is integral to the understanding of the mechanism of these toxins and the development of an effective treatment. Two regions of CfTX-1 have been predicted to have helical structures that are involved with the mechanism of action. Here we have synthesized peptides corresponding to these regions and analyzed their structures using NMR spectroscopy. The peptide corresponding to the predicted N-terminal amphiphilic helix appears unstructured in aqueous solution. This lack of structure concurs with structural disorder predicted for this region of the protein using the Protein DisOrder prediction System PrDOS. Conversely, a peptide corresponding to a predicted transmembrane region is very hydrophobic, insoluble in aqueous solution and predicted to be structured by PrDOS. In the presence of SDS-micelles both peptides have well-defined helical structures showing that a membrane mimicking environment stabilizes the structures of both peptides and supports the prediction of the transmembrane region in CfTX-1. This is the first study to experimentally analyze the structure of regions of a *C. fleckeri* protein.

## 1. Introduction

The Australian box jellyfish, *Chironex fleckeri*, belongs to a family of cubozoan jellyfish that are known for their potent venoms [1,2,3]. In the northern half of the Australian continent, *C. fleckeri* envenomations, ranging from mostly minor to occasionally life threatening, occur frequently, particularly during October to June [4]. Envenomation symptoms include immediate severe pain, cutaneous inflammation, cardiovascular distress and dysfunction, loss of consciousness and potential cardiac arrest [5,6].

The venom of *C. fleckeri* is of particular clinical relevance as rapid cardiovascular collapse followed by death can occur within minutes [6,7]. The majority of the venom toxins are proteins with a variety of distinct functions. These protein toxins include CfTX-like proteins, a variety of enzymes such as proteases and oxido-reductases, CRISP-toxins, an alpha-macroglobulin and a protease inhibitor [8]. The most abundant CfTX protein toxins in the venom are CfTX-1 and -2 and CfTX-A and -B. Both the CfTX-1/2 and CfTX-A/B toxins have potent hemolytic activity (HU_50_ ≤ 161 ng/mL), with the latter toxins being more potent (HU_50_ 5 ng/mL) [9,10,11]. By contrast, the CfTX-1/-2 toxins induce a rapid fatal cardiovascular collapse in rats, whereas CfTX-A and -B have only minor cardiotoxic effects [9]. Phylogenetic analysis of the amino acid sequences of these venom proteins indicates the toxins belong to two functionally and structurally distinct subfamilies; Type I (CfTX-1 and -2) and Type II (CfTx-A and B) [9]. Thus, far, the potent Type I cardiotoxins CfTX-1 and -2 have only been found in *C. fleckeri*, suggesting that these proteins are likely the cause of the rapid cardiovascular collapse observed in severe envenomations and hence, potentially the reason this species is considered the most dangerous of its family [9].

Secondary structure prediction on *C. fleckeri* toxins CfTX-1 and -2 suggests two helical regions might be involved in the mechanism of action [11,12]. These two helical regions comprise an amphiphilic α-helix and transmembrane spanning region. Predicted amphiphilic α-helices in the N-terminal region of toxins from other cubozoan jellyfish (*Chiropsalmus quadrigatus*, *Carybdea rastoni*, *Alatina alata (previously Carybdea alata* [12]), have been suggested to be associated with their hemolytic activity [13,14]. In addition, the predicted transmembrane spanning region consists of a series of highly conserved amino acids in CfTX-1 and -2 as well as the related jellyfish toxins CqTX-A, CrTXs and CaTX-A [11], and has also been implicated in the mechanism of action of these toxins.

Transmembrane spanning regions are commonly seen in pore-forming toxins [15,16], thus suggesting that the cardiotoxicity of CfTX-1 and -2 might result from the transmembrane spanning region integrating itself into the membrane of the cardiomyocytes and creating pores in the process. This would create a rationale for previously observed non-specific ion leakage into the cardiac cell, followed by increased calcium levels. This increase has been suggested to induce irregular contractions of the single cardiomyocyte, leading to a communal flagging of contractions of cardiac cells overall, and thus resulting in cardiovascular collapse [17].

**Figure 1:**
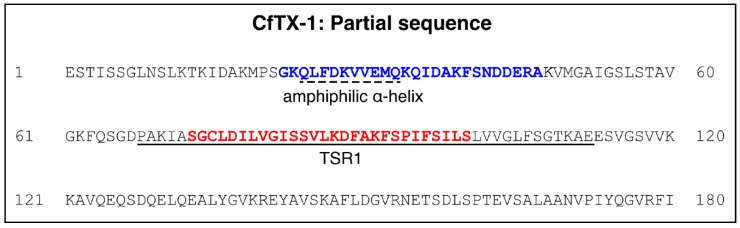
N-terminal region of *C. fleckeri* toxin CfTX-1. The Figure shows 180 out of a total of 436 residues (the N-terminal signal peptide is not included in the Figure). The predicted amphiphilic α-helix and the transmembrane spanning region (TSR1) as defined by Brinkman and Burnell [11] are indicated by a dotted and continuous underline, respectively. The sequences of the two peptides synthesized in the current study include the complete amphiphilic α-helix (blue) and part of the TSR1 (red), respectively.

The locations of the predicted amphiphilic α-helix and transmembrane spanning region in CfTX-1 are shown in Figure 1. There is no experimental evidence for the existence of the putative structures of these two regions and therefore conclusions on the function of these regions remain hypothetic. Experimental evidence on these putative structures is imperative to provide a baseline for future studies directed towards developing effective treatment of *C. fleckeri* envenomation but is also likely to provide insight into the development of bioactive peptides that might have potential for the development of *C. fleckeri* venom derived drugs.

α-Helices have been shown to form in isolation (reviewed in [18]) and can either be derived from larger proteins or present in small naturally occurring peptides such venom derived peptides. The aim of the current study was to determine if regions associated with the predicted helical regions in the CfTX-1 toxin can form well-defined structures in solution. The results of this study provide insight into the autonomous folding capabilities of these regions of CfTX-1 and provide a foundation for future structural analysis on *C. fleckeri* toxins.

## 2. Results

### 2.1. CfTX-1 Peptides—Design and Synthesis

The peptide design was based primarily on the predicted helical regions in CfTX-1 (Figure 1), a 436-residue protein [11]. The length of the predicted amphipathic helix in CfTX-1 involves residues 24–33 whereas the putative transmembrane spanning region with at least one helix, although not precisely defined, was located within residues 68–112. An intermediate length (approximately 25 residues) was chosen for both peptides as peptides of this length are more likely to have secondary structure than smaller peptides (for example see [19]) and are less likely to have significant overlap in the NMR spectra, which can be present in relatively large peptides (for example see [20]). The termini of the synthetic peptides were chosen based on sequence similarity with related toxins including CfTX-2, CqTX-A, CrTXs and CaTX-1 [11]. The sequences of the synthetic peptides, termed CfTX-1_22–47_ and CfTX-1_73–100_ to designate the residue numbering for the N- and C-termini, are shown in Figure 1. Henceforth, the residue numbers for each of the two synthetic peptides will be referred to in numerical order starting with 1, e.g., the first residue of CfTX-1_22–47_ will be referred to as residue 1 of CfTX-1_22–47_.

The peptides were synthesized using Fmoc chemistry, purified using RP-HPLC and characterized using mass spectrometry. CfTX-1_73–100_ was significantly more hydrophobic than CfTX-1_22–47_ based on the retention time on RP-HPLC; 66 min (60% acetonitrile) and 33.5 min (30% acetonitrile) on a 1% min gradient, respectively.

### 2.2. Structural Analysis Using NMR Spectroscopy

**Figure 2:**
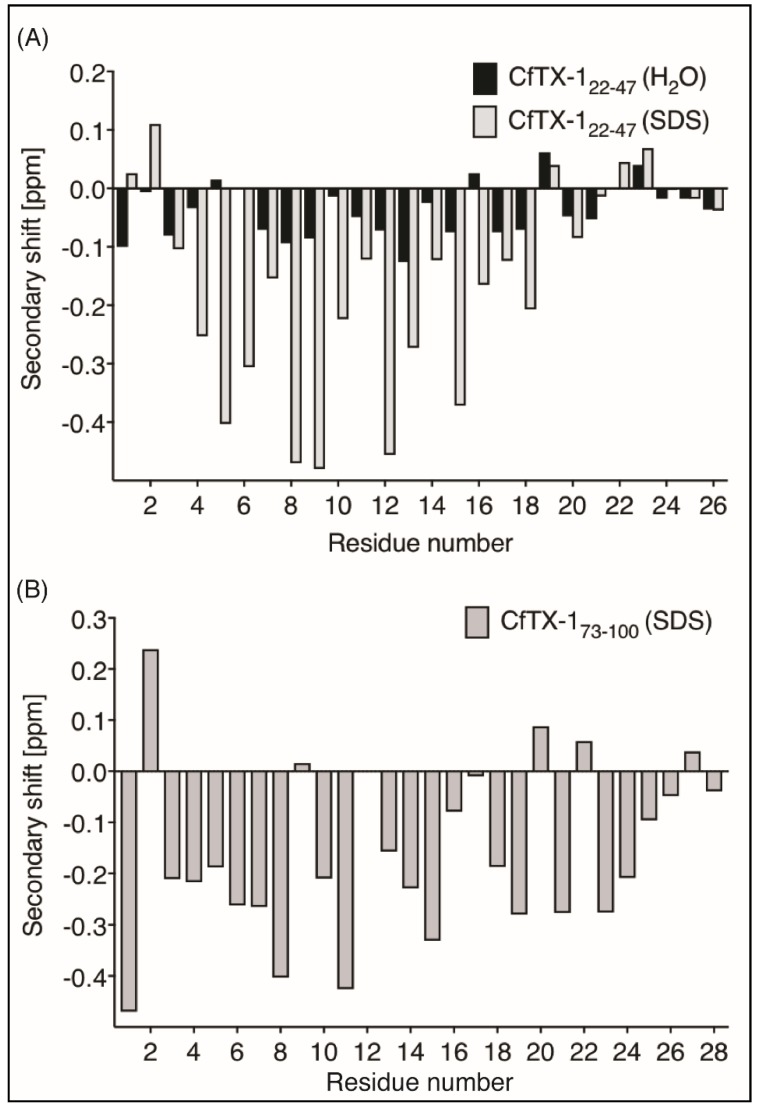
Secondary α-H shifts of CfTX-1_22–47_ and CfTX-1_73–100_. (**A**) Secondary α-proton shifts for CfTX-1_22–47_ in H_2_O (black bars) and in 100 mM SDS (grey bars with black border). Secondary shift values of CfTX-1_22–47_ in an aqueous environment are generally within ±0.1, indicating lack of structure; whereas the values of CfTX-1_22–47_ in SDS are lower than −0.1 ppm between residues 4 and 19 (corresponding to residues 25 and 40 in the full-length protein), indicating an α-helical structure in this environment. (**B**) Secondary α-proton shifts for CfTX-1_73–100_ in SDS. All secondary shift values between residues 3 and 20 (corresponding to residues 75 and 92 in the full-length protein), except residue 9, 12 and 17 (corresponding to residues 81, 84 and 89 in the full-length protein) are below −0.1 ppm indicating a helical structure. Residue 12 (corresponding to residue 84 in the full-length protein) could not be assigned due to overlap with other NOEs.

NMR spectroscopy was used for the structural analysis of the two peptides. The one-dimensional spectra of CfTX-1_22–47_ had limited chemical shift dispersion in the amide region. The sharp peaks in the NMR spectra suggest that the peptide was present in a monomeric conformation, but a quantitative analysis was not carried out. Resonances were assigned using two-dimensional TOCSY and NOESY spectra and the secondary shifts were calculated by subtracting random coil shifts [21] from the αH shifts. The secondary shifts for CfTX-1_22–47_ are close to random coil values as shown in Figure 2, indicating that the peptide lacks structure in an aqueous environment.

CfTX-1_73–100_ was insoluble in aqueous solution but soluble in 20% acetonitrile. However, the one-dimensional spectrum of CfTX-1_73–100_ in 20% acetonitrile showed broad peaks, indicating that the peptide might be aggregating under these conditions, but other factors could also be involved. Given the potential aggregation of CfTX-1_73–100_ and the lack of structure for CfTX-1_22–47_, the NMR data was therefore repeated for both peptides in the presence of deuterated sodium dodecyl sulfate (SDS) micelles. In SDS, both peptides had enhanced chemical shift dispersion in the amide region and the secondary shifts of CfTX-1_22–47_ and CfTX-1_73–100_ were consistent with helical structure based on the consecutive negative shifts (more negative than −0.1) [22] as shown in Figure 2.

**Figure 3:**
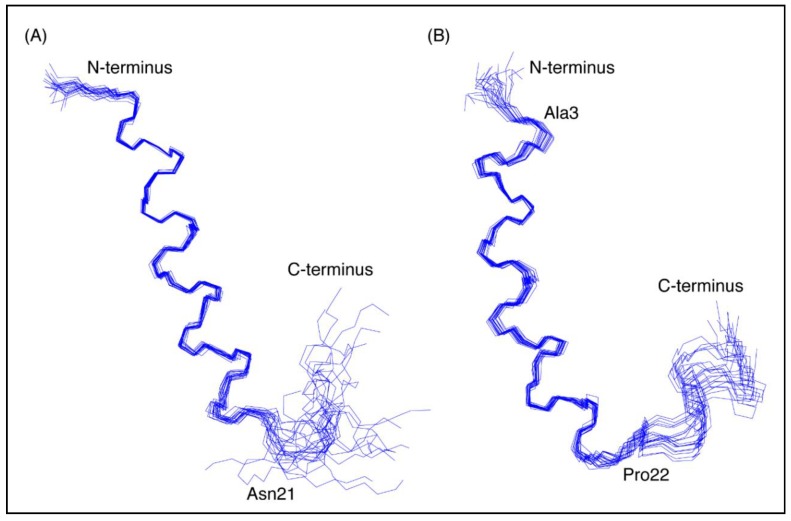
Three-dimensional structures of the CfTX-1 derived peptides based on data recorded in SDS-micelles. The 20 lowest energy structures of (**A**) CfTX-1_22–47_ and (**B**) CfTX-1_73–100_ are shown highlighting the well-defined helical region between residues 4–19 and 3–20 (corresponding to residues 25–40 and 75–92 in the full-length protein), respectively, as well as the less defined C-terminus. In CfTX-1_73–100_ Cys3 (corresponding to Cys75 in the full-length protein) was substituted for an alanine residue (Ala3) to prevent disulfide bond formation. The locations of Asn21 and Pro22 (corresponding to Asn42 and Pro94 in the full-length protein) are labeled in CfTX-1_22–47_ and CfTX-1_73–100_, respectively, and correspond to the start of the C-terminal tails that are more disordered than the helical regions. The Figure was created in MOLMOL [24].

**Figure 4:**
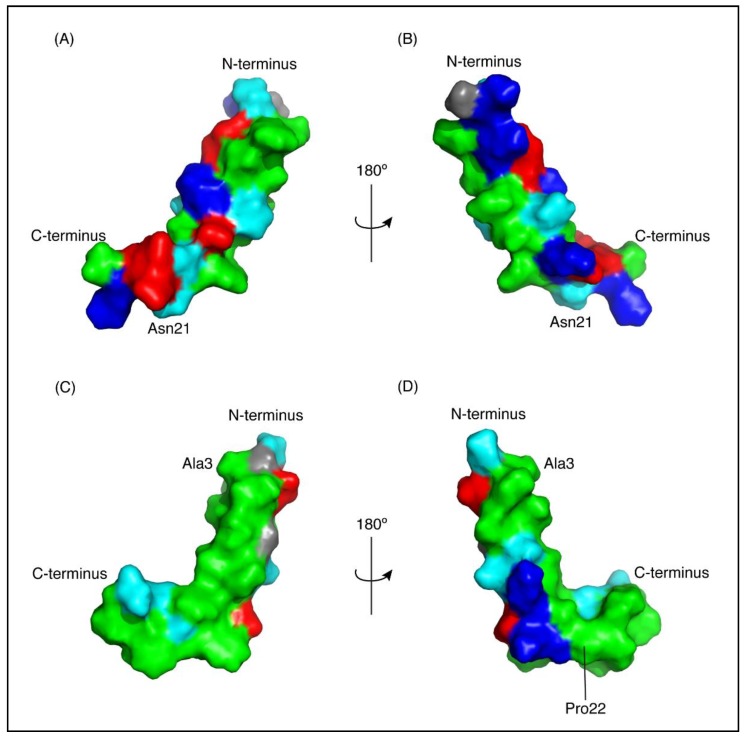
Surface representation of CfTX-1 derived peptides in an SDS-micelles environment. Hydrophobic residues are represented in green, polar residues in cyan, glycine residues in grey, positively and negatively charged residues in dark blue and red, respectively. The charge states represent expected states at physiological conditions based on generic pKa values for glutamic acid and aspartic acid residues (~4) and lysine and arginine residues (>10). CfTX-1_22–47_ (**A**,**B**) has a cluster of positively and negatively charged residues on one face of the molecule and a cluster of hydrophobic residues on the other face. CfTX-1_73–100_ (**C**,**D**) shows an extended patch of hydrophobic residues. The arrow pivoting around the axis in the top and bottom center represents the 180° turn between Figure (**A**,**C**) and (**B**,**D**), respectively. The Figure was generated in PyMol (Schrödinger, Inc., New York, NY, USA).

The three-dimensional structures of both peptides, in the presence of SDS, were determined using the program CYANA [23]. The structures of CfTX-1_22–47_ were determined based on 263 NOEs and 43 dihedral angle restraints. Secondary structure analysis using MOLMOL [24] indicated that an α-helix was present between residues 4 to 19 (corresponding to residues 25 to 40 in the full-length protein). The structures of CfTX-1_73–100_ were determined based on 437 NOEs and 45 dihedral angle restraints. Secondary structure analysis using MOLMOL indicated that an α-helix was present between residues 3–20 (corresponding to residues 75–92 in the full-length protein). The structural statistics for the peptides are given in Table 1 and an overlay of the 20 lowest energy structures given in Figure 3. The greater number of NOEs for CfTX-1_73–100_ appears to be related to more overlap in the amide region of CfTX-1_22–47_. A surface structure representation for both peptides is given in Figure 4.

### 2.3. Structure Predictions for CfTX-1

**Figure 5:**
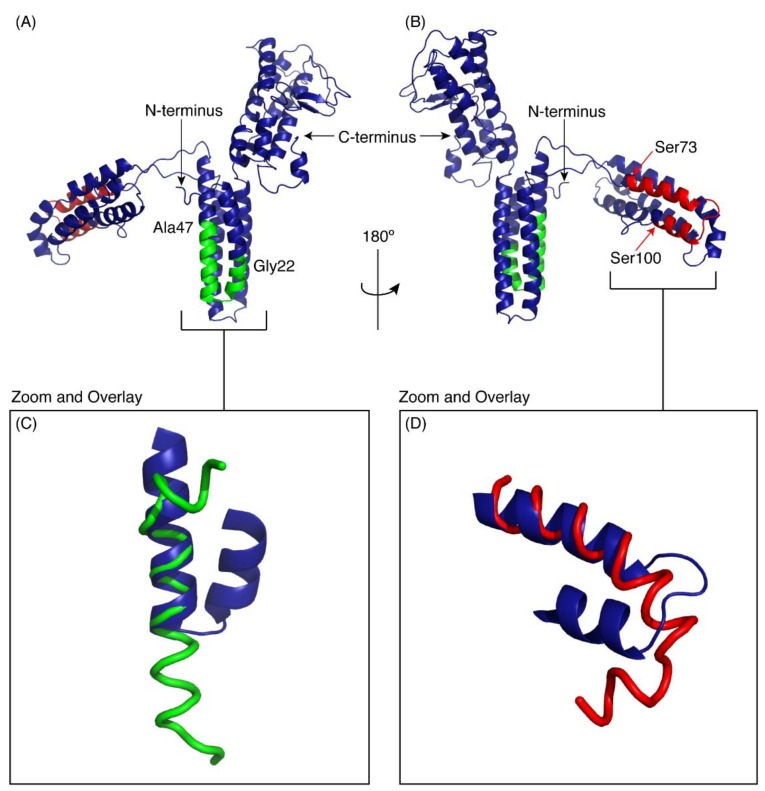
I-TASSER model of CfTX-1. The model indicates three multi-helical regions separated by loop structures. The location of CfTX-1_22–47_ is shown in green and CfTX-1_73–100_ in red. (**A**,**B**) are rotated 180° with respect to each other. Panels C and D show the overlay of the experimental peptide structures for CfTX-1_22–47_ (**C**) and CfTX-1_73–100_ (**D**) with the corresponding regions in the model of CfTX-1. The model is based on the crystal structure of an insecticidal δ-endotoxin Cry8Ea1 from *Bacillus thuringiensis* (PDB code 3EB7). The Figure was generated in PyMol (Schrödinger, Inc., New York, NY, USA).

As there is no experimental structure available for CfTX-1 we used I-TASSER [25] to produce a model to compare our peptide structures with the predicted structure of the full-length protein. Five models of CfTX-1 were generated by I-TASSER and the highest-ranking protein structure with a C-score of −3.12 is shown in Figure 5. I-TASSER outputs C-scores ranging from −5 to 2, with higher scores representing higher confidence in the model [26]. Although −1.5 has been used previously as a cutoff for confidence in models, there can still be significant modeling accuracy in models with C-scores below this limit [26]. The structure was based on the crystal structure of an insecticidal δ-endotoxin Cry8Ea1 from *Bacillus thuringiensis* (PDB code 3EB7), which has 25% sequence identity with CfTX-1. The first 373 residues of the model are dominated by α-helices whereas the remaining 83 residues are a mix of helices, coil structures, bends and β-sheets. The top threading templates used in the modelling are given in Supplementary Table S1 and the structural analogues in the PDB are given in Supplementary Table S2. The structural analogues include proteins involved in transport, and enzymes such as RNA polymerase.

The regions corresponding to the peptides CfTX-1_22–47_ and CfTX-1_73–100_ are shown on the model in green and red respectively. Panels C and D show the overlay of the experimental peptide structures with the corresponding regions in the model of CfTX-1. CfTX-1_22–47_ has an extended helical region compared to the model of the full-length protein. By contrast, the structure of CfTX-1_73–100_ is similar to the modelled structure, but the “bend” at the C-terminus of CfTX-1_73–100_ rotates the C-terminal residues into a slightly different orientation from that present in the CfTX-1 model.

**Figure 6:**
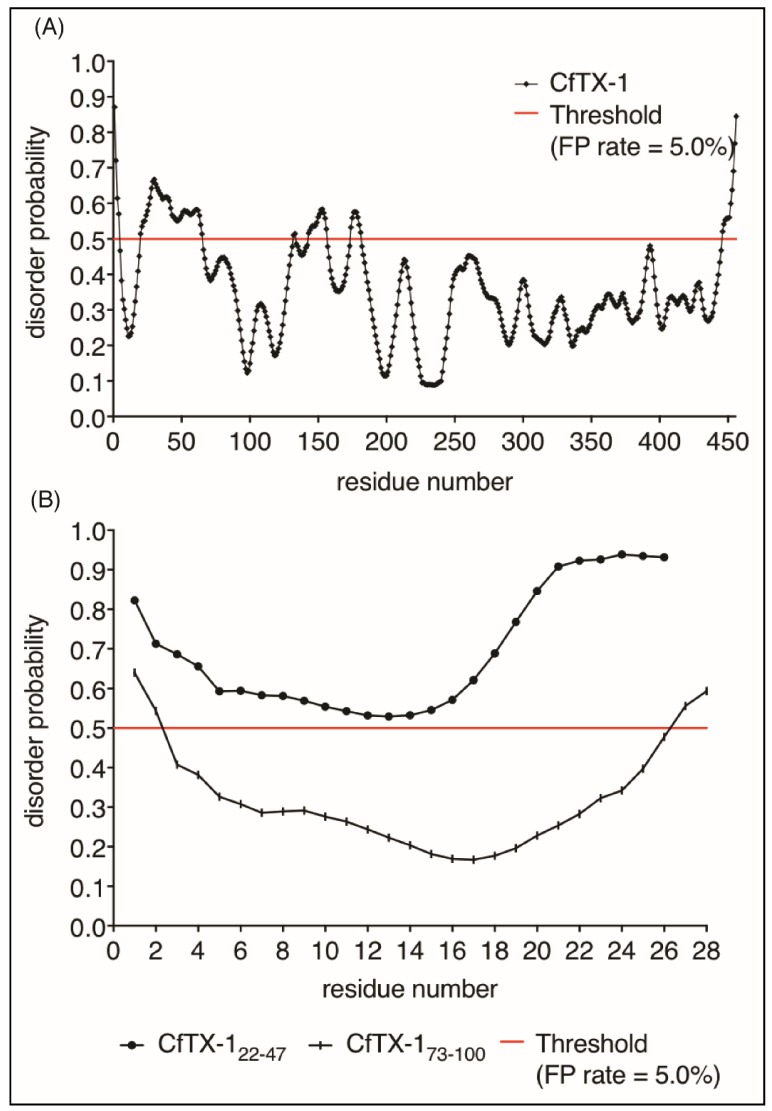
Protein disorder predictions. Residues above/below the threshold probability of 0.5 (red line) are classed as disordered/structured with a false positive rate of 5.0%. (**A**) The disorder prediction for CfTX-1, which indicates a mostly structured protein with four disordered regions between residues 20–65 (location of CfTX-1_22–47_), 131–132, 143–156 and 174–181. (**B**) This disorder prediction for CfTX-1_22–47_ and CfTX-1_73–100._ The former appears to lack structure whereas the latter appears structured between residues 3–26 (corresponding to residues 75–98 in the full-length protein). The analysis was conducted with the online protein disorder prediction system PrDOS [27].

Given the lack of structure of CfTX-1_22–47_ in aqueous solution we analyzed the propensity of CfTX-1 to have disordered structure using the program PrDOS [27]. This analysis indicated that the majority of the protein is structured (Figure 6), consistent with the I-TASSER model. Interestingly, there are small regions predicted to be disordered including residues 20–65 which encompasses the sequence of CfTX-1_22–47_. The isolated sequence of CfTX-1_22–47_ was also predicted to be disordered by PrDOS (Figure 6A). By contrast, CfTX-1_73–100_ was predicted to be structured (Figure 6B). The NMR data for CfTX-1_22–47_ solubilized in water is consistent with the PrDOS results, which indicated that the respective sequence is unstructured.

## 3. Discussion

This study provides the first experimental data regarding the structures of peptides derived from the putatively pore forming *C. fleckeri* toxin, CfTX-1. Two of the regions previously predicted to be involved in helices [11] behave differently in aqueous solution but both form well defined helices in the presence of SDS. Structural predictions of the full-length protein provided some context for the experimental structures.

The region predicted to form an amphiphilic α-helix in CfTX-1 (residues 25–32) appears to be disordered in aqueous solution based on the chemical shift analysis of CfTX-1_22–47_. By contrast, the chemical shift analysis in the presence of 100 mM SDS has several consecutive negative shifts, consistent with helical structure. This helical structure was confirmed by determination of the three-dimensional structure (Figure 2). The helix formed in the presence of SDS has an amphiphilic nature as one face contains mainly charged residues whereas the opposite face is significantly more hydrophobic. The transition of unstructured to helical structure in the presence of membranes or membrane-mimicking environments has previously been shown for other peptides, such as the antimicrobial peptides MSI-78 and magainin-2 [28].

The model of CFTX-1 predicted using I-Tasser shows that residues 22–47 are involved in a helical structure (including a turn centered at residue 33) but analysis of the intrinsic disorder using PrDOS suggests that this region is unstructured in the full-length protein, as well as in the isolated peptide. Our NMR results in aqueous solution are consistent with the PrDOS results; however, the propensity of the peptide to form helical structure, albeit in the presence of a detergent, is consistent with the modelled structure and the predicted secondary structure [11]. The I-Tasser model of CfTX-1 and the PrDOS results appear conflicting, but as the I-TASSER model ranks on the lower end of the C-score-scale (−3.12 on a scale of −5 to 2) it is possible that the model is not reliable for residues 22–47. An experimental structure of the full-length protein is required to determine whether this region is structured or not, and whether or not it undergoes a structural reorganisation depending on the environment.

The region of CfTX-1 predicted to be transmembrane spanning [11,12] forms a helical hairpin structure in the model, a structure commonly found in pore forming toxins [29]. The original prediction only referred to this region as transmembrane spanning, as there was no three-dimensional information to suggest a hairpin structure. CfTX-1_73–100_ encompasses many of the residues predicted to be involved in a transmembrane region in CfTX-1. CfTX-1_73–100_ is significantly more hydrophobic than CfTX-1_22–47_, is insoluble in aqueous solution and appears to aggregate in a water/acetonitrile solution. However, in the presence of SDS the peptide is soluble, gives relatively sharp peaks in the NMR spectra, and has a well-defined helical structure for most of the peptide. Our experimental structure of CfTX-1_73–100_ in SDS is largely consistent with the model of CfTX-1. However, the orientation of the turn region of CfTX-1_73–100_ differs slightly from the model. Furthermore, the terminal regions of the peptide are disordered, as is often present in small, unconstrained peptides. Overall, our analyses on CfTX-1_73–100_ are consistent with this region of the protein being involved in membrane spanning [11], given that transmembrane peptides are generally hydrophobic, aggregate in aqueous solutions and form helices under certain conditions [30], similar to what we have observed for CfTX-1_73–100_. Based on our structural data it is interesting to speculate that this helical region is involved with the putative pore formation that appears to be associated with *C. fleckeri* toxins [17,31]. However, further research into the structure of *C. fleckeri* toxins and their mechanism is required to draw a definitive conclusion.

In summary, this study represents the first structural characterization of isolated regions of CfTX-1. Amphiphilic helices have been predicted for the N-terminal region of several jellyfish toxins, including CfTX-1, but the present data suggest this region does not have an intrinsic propensity to form an α-helix in isolation. By contrast, the experimental data are consistent with the structural predictions for the putative transmembrane spanning region. The current study also provides information on the chemical and physical properties of these peptides, which might facilitate the development of effective treatment and/or venom-derived drug development. More research into the structures and bioactivity of the full-length protein is needed to elucidate the mode of action of *C. fleckeri* toxins.

## 4. Materials and Methods

### 4.1. Peptide Synthesis and Purification

Peptides were synthesised by solid-phase peptide synthesis on an automated PS3™ peptide synthesizer (Protein Technologies Inc., Tucson, AZ, USA) using fluorenylmethyloxycarbonyl (Fmoc) chemistry on a 0.1 mmole scale. Peptides were assembled on 2-chlorotrityl chloride resin (Auspep, Melbourne, Australia). Amino acids were activated in peptide grade dimethylformamide (DMF, Auspep, Melbourne, Australia) using 2-(1H-benzotriazol-1-yl)-1,1,3,3-tetramethyluronium hexafluorophosphate (HBTU, Iris Germany, München, Germany). The first amino acid was coupled manually. Peptides were cleaved in a trifluoroacetic acid (TFA)/H_2_O/triisopropylsilane (TIPS) (95:2.5:2.5) mixture for 2 h. After cleavage, the TFA was evaporated with nitrogen and the peptides were precipitated in cold diethyl ether (4 °C). The ether was removed by filtration and the precipitated peptides were solubilised in a mixture of acetonitrile (acetonitrile)/H_2_O/TFA (CfTX-1_22–47_: 5:94.95:0.05; CfTX-1_73–100_: 25:74.95:0.05) and then lyophilised. The resulting peptides were purified by reversed phase high performance liquid chromatography (RP-HPLC) (Agilent 1200 Infinity series, Agilent Technologies, Inc., Santa Clara, CA, USA) on a semi-preparative C-18 column (Jupiter 4 μm C_18_ Proteo 90 A° 250 mm × 10.00 mm, Penomenex, Inc., Torrance, CA, USA). The peptides were eluted using a 1%/min gradient of solvent B (solvent A: 0.05% TFA, solvent B: 90% acetonitrile, 0.05% TFA) starting at 0% and 25% solvent B for CfTX-1_22–47_ and CfTX-1_73–100_, respectively and finishing at 90% solvent B. Absorbance traces of the eluent were collected at 214 and 280 nm. The purity of the eluted peptide was verified by analytical RP-HPLC (Agilent 1260 Infinity series, Agilent Technologies, Inc., Santa Clara, CA, USA) with a C-18 column (Eclipse Plus 3.5 μm 4.6 mm × 100 mm, Agilent Technologies, Inc., Santa Clara, CA, USA) and the mass was analysed using MALDI mass spectrometry.

### 4.2. NMR Spectroscopy and Structure Determination

The purified peptides were dissolved in a mixture of 89.9% H_2_O:10%D_2_O:0.1%TFA and 69.9% H_2_O:20% acetonitrile: 10%D_2_O:0.1%. A 600 MHz AVANCE III NMR spectrometer (Bruker, Karlsruhe, Germany) with a cryogenically cooled probe was used to acquire two-dimensional (2D) ^1^H-^1^H TOCSY and ^1^H-^1^H NOESY spectra at 303 K. 4,4-dimethyl-4-silapentane-1-sulfonic acid was used as a chemical shift reference. NMR spectra were also collected for both peptides in 100 mM SDS 90% H_2_O:10%D_2_O. All spectra were recorded with an interscan delay of 1 s. NOESY spectra were acquired with mixing times of 200–300 ms, and TOCSY spectra were acquired with isotropic mixing period of 80 ms. Standard Bruker pulse sequences were used with an excitation sculpting scheme for solvent suppression. Assignments were made based on the procedure described by [32]. Slowly exchanging amide protons were detected by acquiring a series of one-dimensional and TOCSY spectra after dissolution of the peptides in 100 mM SDS in D_2_O.

Three-dimensional structures were determined with the program CYANA [23]. CYANA is a program that calculates the structure of biological macromolecules, such as peptides and proteins, in an automated process that is based on conformational constraints obtained from NMR-analysis. Non-intra residue peaks in the NOESY spectra were automatically assigned and an ensemble of structures calculated. Dihedral angle restraints derived from TALOS+ [33] were used in the structure calculations. TALOS+ is a hybrid method for predicting protein backbone torsion angles by establishing an empirical relation between ^13^C, ^15^N and ^1^H NMR chemical shifts and backbone torsion angles ϕ and ψ. Hydrogen bonds predicted from preliminary structures, that were consistent with the slowly exchanging amide protons, were subsequently included in the structure calculations. A final ensemble of 100 structures was calculated and the 20 structures with the lowest target functions chosen to represent the structures of CfTX-1_22–47_ and CfTX-1_73–100_. The structure statistics were calculated with CYANA and MOLMOL [24].

### 4.3. Structural Predictions for CfTX-1, CfTX-1_22–47_, CfTX-1_73–100_

I-TASSER [25] was used to generate a three-dimensional structure model of CfTX-1. I-TASSER is an online platform for protein structure and function predictions. I-TASSER operates in a three-step process. First, the server searches for template proteins of similar folds from the protein data bank library by LOMETS (Local Meta-Threading-Server), which is a locally installed meta-threading approach. Secondly, full-length models are reassembled from the continuous fragments excised from the PDB templates by replica-exchange Monte Carlo simulations with unaligned regions such as loops built by ab initio modeling. Thirdly, the fragment assembly simulation is performed again and the lowest energy structures are selected. The final full-atomic models are obtained from the REMO-algorithm, which builds the atomic elements from the selected iTASSER decoys through the optimization of the hydrogen-bonding network.

PrDOS [27] was used to predict natively disordered regions of CfTX-1, CfTX-1_22–47_ and CfTX-1_73–100_. PrDOS is a protein disorder prediction system that is based on two predictors: (1) the amino acid sequence of the protein being analysed (implemented using support vector machine) and (2) template proteins or homologues with available structural information (implemented using PSI-BLAST (Position-Specific Iterated)) and PrDOS measure of disorder. The method was assessed by the CASP benchmark and achieved high performance, in particular for short disordered regions.

## Figures and Tables

**Table 1:** Structural statistics for CfTX-1_22–47_ and CfTX-1_73–100_ based on NMR data recorded in 100 mM SDS_._

Parameter	Peptide	
	CfTX-1_22–47_	CfTX-1_73–100_
Experimental Restraints		
Interproton distance restraints	263	437
*Intraresidue*, *|i* − *j|* = 0	122	145
*Sequential*, *|i* − *j|* = 1	82	158
*Medium range*, 1 < *|i* − *j|* < 5	59	134
Dihedral-angle restraints	43	45
**R.M.S. Deviations from Mean Coordinate Structure (Å)**		
Backbone atoms (indicated)	0.32 ± 0.12 (res. 4–19)	0.43 ± 0.17 (res.3–20)
All heavy atoms (indicated)	1.48 ± 0.23 (res. 4–19)	1.06 ± 0.25 (res. 3–20)
Backbone atoms (all)	2.19 ± 0.79	0.89 ± 0.32
All heavy atoms (all)	3.18 ± 0.87	1.36 ± 0.32
**Ramachandran (%)**		
Residues in most favoured regions	85.8	91.3
Residues in additionally allowed regions	14.2	8.7

## Supplementary Materials

The following are available online at http://www.mdpi.com/1660-3397/16/6/201/s1, Table S1: Top 10 threading templates used by I-TASSER, Table S2: Top 10 identified structural analogues in PDB.
