# Supplementary material for: Structural Characterisation of Predicted Helical Regions in the Chironex fleckeri CfTX-1 Toxin
Source: Mar Drugs. 2018 Jun 7;16(6):201. doi: 10.3390/md16060201 (PMC6024933; doi:10.3390/md16060201)
Supplement: Supplementary file 1 [file marinedrugs-16-00201-s001.pdf]

Supplementary Table S1: Top 10 threading templates used by I-TASSER

Rank	PDB Hit	Iden1	Iden2	Coverage	Norm. Z-score
**1**	3eb7A	0.15	0.17	0.73	1.25
**2**	3zkvA	0.11	0.18	0.68	1.35
**3**	5ic0A	0.07	0.17	0.90	1.74
**4**	4c0oA	0.13	0.16	0.66	1.35
**5**	3eb7A	0.12	0.17	0.77	1.27
**6**	5j65A	0.11	0.17	0.72	1.73
**7**	1dlcA	0.11	0.18	0.75	1.17
**8**	3ay5A	0.09	0.14	0.54	1.29
**9**	2qkgA	0.12	0.17	0.77	1.27
**10**	5jliA	0.08	0.13	0.64	1.23

a) Ident1 is the percentage sequence identity of the templates in the threading aligned region with the query sequence.
b) Ident2 is the percentage sequence identity of the whole template chains with query sequence.
c) Cov represents the coverage of the threading alignment and is equal to the number of aligned residues divided by the length of query protein.
d) Norm. Z-score is the normalized Z-score of the threading alignments. Alignment with a Normalized Z-score >1 mean a good alignment and vice versa.

Supplementary Table S2: Top 10 identified structural analogues in PDB

Rank	PDB Hit	TM-score	RMSD[a]	Iden[a]	Coverage
**1**	5ic0A	0.877	1.4	0.061	0.895
**2**	2x0cA	0.389	4.12	0.050	0.450
**3**	4fhmB	0.375	7.02	0.034	0.605
**4**	1i3qB	0.372	7.32	0.040	0.612
**5**	3wajA	0.370	7.81	0.044	0.656
**6**	5a9q1	0.369	6.79	0.066	0.572
**7**	5flmB	0.368	7.18	0.061	0.594
**8**	3f7fD	0.365	6.83	0.043	0.581
**9**	4qiwB	0.361	7.21	0.051	0.579
**10**	5anbK	0.360	7.57	0.039	0.621

a) Ranking of proteins is based on TM-score of the structural alignment between the query structure and known structures in the PDB library. TM-score is a metric for determining the similarity of two protein structure models based on their given residue equivalency.
b) RMSD[a] is the root-mean-square deviation (RMSD) between residues that are structurally aligned by TM-align.
c) Iden[a] is the percentage sequence identity in the structurally aligned region.
d) Coverage represents the coverage of the alignment by TM-align and is equal to the number of structurally aligned residues divided by length of the query protein.
